# Effect of Synthesis Temperature on the Size of ZnO Nanoparticles Derived from Pineapple Peel Extract and Antibacterial Activity of ZnO–Starch Nanocomposite Films

**DOI:** 10.3390/nano10061061

**Published:** 2020-05-30

**Authors:** Hasbullah Hassan Basri, Rosnita A. Talib, Rashidah Sukor, Siti Hajar Othman, Hidayah Ariffin

**Affiliations:** 1 Department of Process and Food Engineering, Faculty of Engineering, Universiti Putra Malaysia, Serdang 43400, Malaysia; hasbullah.hassanbasri08@gmail.com (H.H.B.); s.hajar@upm.edu.my (S.H.O.); 2 Halal Products Research Institute, Universiti Putra Malaysia, Serdang 43400, Malaysia; 3 Department of Food Science, Faculty of Science and Food Technology, Universiti Putra Malaysia, Serdang 43400, Malaysia; rashidah@upm.edu.my; 4 Institute of Tropical Agriculture and Food Security, Universiti Putra Malaysia, Serdang 43400, Malaysia; 5 Institute of Tropical Forestry and Forest Products, Universiti Putra Malaysia, Serdang 43400, Malaysia; hidayah@upm.edu.my

**Keywords:** pineapple peel, synthesis temperature, ZnO NPs, starch, nanocomposite films

## Abstract

This research investigated the effect of synthesis temperature on the size and shape of zinc oxide (ZnO) nanoparticles (NPs) synthesized using pineapple peel waste and antibacterial activity of ZnO NPs in starch films. Zinc oxide NPs synthesized at different temperatures were characterized by Fourier transform infrared spectroscopy, X-ray diffraction analysis, field-emission scanning electron microscopy, energy-dispersive X-ray spectroscopy, and transmission electron microscopy. Micrographs of ZnO NPs synthesized at 28 and 60 °C showed that synthesis temperature affected the sizes and shapes of ZnO NPs. The non-heated (28 °C) condition resulted in NPs with diameters in the range of 8–45 nm with a mixture of spherical and rod shapes, whereas the heated (60 °C) condition led to NPs with diameters in the range of 73–123 nm with flower rod shapes. The ZnO–starch nanocomposite films incorporated with 1, 3, and 5 wt.% ZnO NPs were prepared via a film casting method. The antibacterial activity of the films against Gram-positive and Gram-negative bacteria was investigated using the disc diffusion method. The results showed an increase in the inhibition zone for Gram-positive bacteria, particularly *Bacillus subtilis*, when the concentration of ZnO NPs incorporated in the film was increased from 1 to 5 wt.%.

## 1. Introduction

Zinc oxide (ZnO) has drawn the attention of numerous researchers because of its morphology and its significant antibacterial/antifungal activity toward various bacterial/fungal species [1]. In addition, ZnO has numerous biological applications, because it is environmentally friendly, easy to prepare, non-toxic, bio-safe, and biocompatible [2]. Various physical and chemical methods can be used to synthesize ZnO.

In recent years, researchers have actively investigated the green synthesis approach, which is an environmentally friendly method that is safe for materials that contact food and biomedical applications, where it generates fewer adverse effects than physical and chemical methods [3]. The numerous methods for ZnO NPs production, including hydrothermal synthesis, precipitation the microemulsion method, vapor deposition and the sol-gel process, enables obtaining particles with a variety of structures, sizes, and shapes [4,5,6]. According to Ma et al. [7], the simplest route is an acid–base precipitation method which was widely used to obtain ZnO nanoparticles for either biological or coating application. An aqueous precipitation process was employed using precursors, such as zinc acetate (Zn(CH_3_COO)_2_) [8], zinc nitrate (Zn(NO_3_)_2_) [9] or zinc sulfate (ZnSO_4_) [10], and an alkaline aqueous solution, such as sodium hydroxide (NaOH), are usually prepared in deionized/distilled water.

Zinc oxide nanoparticles incorporated with organic compounds, such as starch [11,12,13], methylcellulose (MC) [14], acrylic binder [15], alginate [16], polyimide [17], and chitosan [18], for nanocomposite coating in numerous industrial sectors, including food, packaging, textiles, environmental, and health care, showed strong antibacterial activity, dielectric properties, and UV absorbance. Zinc oxide NPs incorporated into the starch-based matrix, because the starch molecules could control the ZnO particle growth, coagulation, and flocculation. The ZnO particles were unable to move freely and dispersed uniformly in the nano-network [7]. According Nafchi et al. [11], incorporation of zinc oxide nanorods with sago starch and bovine gelatin bionanocomposite have potential as an active packaging material for food, because the films exhibited UV absorption and an excellent antibacterial activity against *Escherichia coli*. The more ZnO NPs concentration added into the tapioca starch film increased the efficacy of the film to inhibit pathogenic bacteria of *E. coli* and *Salmonella* sp. [19].

Inorganic nanoparticles including silver (Ag) [20,21,22], titanium oxide (TiO_2_) [23,24,25], zinc oxide (ZnO) [26,27,28], iron oxide (Fe_3_O_4_) [29], copper oxide (CuO) [30,31,32], magnesium oxide (MgO) [25,32,33], nitric oxide (NO) [34,35], and aluminum oxide (Al_2_O_3_) [32,36] have been extensively studied for their antimicrobial activities. Certain metal oxide nanoparticles have been applied in food industry packaging and coatings because of their antibacterial and antifungal properties such as CuO [37,38], Ag [39,40], and ZnO [41,42]. Nanoparticles (NPs) have been shown to have a positive result of antibacterial activities against both Gram-positive and Gram-negative bacteria.

In green synthesis approaches, plant extracts have been reported to be advantageous over microbes, because they do not require high isolation and cultivation costs. Furthermore, high yields can be obtained in a short period, making the method potentially suitable for industrial-scale production [43]. Plant extract is used as a potential substitute for reducing and stabilizing agents in green synthesis because of its combination of biological components such as alkaloids, terpenoids, tannins, phenolics, amino acids, proteins, enzymes, polysaccharides, and vitamins [44]. Different parts of plant extracts have been used for synthesizing ZnO NPs including *Rosa canina* fruit extract [45], *Aloe vera* leaf extract [46], *Trifolium pratense* flower extract [47], *Hibiscus rosa sinensis* [48], leaf extract of *Azadirachta indica* [49], *Physalis alkekengi* L. seed extract [50], leaf extract of *Camellia sinensis* [51], dried sap of roots and shoots of *Astragalus gummifer* [52], aqueous extract of *Abutilon indicum* [53], *Zea mays* leaf extract [54], and aqueous extract of *Dittrichia graveolens* [55].

In addition, various fruit peel extracts are attracting interest because they have properties similar to those of plant extracts; certain fruit peels are also rich in the aforementioned biocomponents and are readily available [56]. The ability to obtain NP-based products via green synthesis using fruit peels is in high demand because fruit peels usually end up as solid waste. Abandoned fruits and their waste can cause severe problems in the environment because they can accumulate in agro-industrial yards without being properly treated. Disposal of these wastes can be expensive because of the high cost of transportation and the scarcity of landfills, resulting in the wastes eventually posing a threat to the environment [57]. The preparation of ZnO NPs from fruit peels utilizes peel waste, thereby mitigating environment-related problems.

The synthesis of metal oxide NPs from the peels of fruits such as banana [58], *Citrus sinensis* [59], jackfruit [60], lemon [61], mango [62], *Musa paradisiacal* [63], pomegranate [64], tangerine [65], *Punica granatum* [66], *Garcinia mangostana* [67], *Citrus aurantifolia* [68], and *Nephelium lappaceum* [69] has already been reported, and numerous studies have examined this approach. According to the reported information, banana peel extract is a potential material for the synthesis of Ag NPs with good antimicrobial activity against certain pathogenic microorganisms [58]. *Punica granatum* peel extract was successfully used to produce ZnO NPs by functioning as a reducing and stabilizing agent during the synthesis process, and the obtained ZnO NPs were effective in inhibiting the growth of bacteria [66].

Pineapple, scientifically known as *Ananas comosus* and a member of the family Bromeliaceae, is widely used in the food and beverage industries because of its naturally sweet and sour taste. Because of its wide range of applications in the food industry, such as in drinks, purees, pastes, and jams, tons of solid pineapple waste are generated each year, creating disposal problems. According to Shamsudin et al. [70], pineapple peels represent approximately 37.1% of the total fruit. These peels are rich in phytochemical compounds from which bioactive compounds such as ZnO can be extracted.

To the best of our knowledge, the synthesis of ZnO NPs from pineapple peel waste has not been previously reported. Therefore, in the present work, ZnO NPs were synthesized from pineapple peel extract and the effect of the synthesis temperature on the formation of ZnO NPs was studied. The ZnO NPs were characterized using various spectral analyses, and the antibacterial efficacy of ZnO–starch nanocomposite films was evaluated via the disc diffusion method. We found that the zone of inhibition for Gram-positive bacteria *Bacillus subtilis* increased when the concentration of ZnO NPs incorporated in the film was increased from 1% to 5%.

## 2. Materials and Methods

### 2.1. Raw Materials

The pineapple peel waste was obtained from local fruit stalls in Sri Serdang, Serdang, Selangor. Cassava starch (Brand Kapal ABC, George Town, Malaysia) was purchased from a mini-market in Sri Serdang. Analytical grade chemicals were used in all experiments including synthesis of ZnO nanoparticles and media preparation for growth of bacterial cells. Zinc nitrate hexahydrate (Zn(NO_3_)_2_·6H_2_O, 98% purity) was purchased from Sigma–Aldrich (St. Louis, MO, USA) and used as a precursor to synthesize ZnO NPs. Glycerol and sodium hydroxide (NaOH) was purchased from R&M Marketing (UK). The bacterial cultures of Gram-negative bacteria *Salmonella enterica* serovar Choleraesuis and Gram-positive bacteria *Bacillus subtilis* UPMC1175 and antibiotics (Streptomycin) was obtained from the Institute of Bioscience Universiti Putra Malaysia.

### 2.2. Preparation of Pineapple Peel Extract

The fresh pineapple peels were initially washed with water and then rinsed three times with deionized water. The pineapple peels were dried in an oven at 60 °C overnight [71]. The mass of each sample was recorded before and after drying. Dried peels were chopped into small pieces and ground using a mechanical grinder to yield a coarse powder (to increase the specific surface area of the material) and stored in airtight plastic bottles at room temperature (28 °C). Then, 10 g of the powder was boiled in a conical flask in 100 mL of distilled water for 15 min to produce pineapple peel solutions [2]. The aqueous extract was allowed to cool to room temperature and was subsequently filtered using Whatman No. 1 filter paper. A clear filtrate was collected and stored at 4 °C until further use.

### 2.3. Preparation of ZnO NPs

The ZnO NPs were synthesized at two different temperatures, 60 °C and non-standard room temperature, 28 °C (refers as the measured room temperature). The synthesis temperature was monitored using a thermometer. Briefly, 50 mL of 0.01 M Zn(NO_3_)_2_·6H_2_O was added to 1 mL of pineapple peel extract. The pH of the solution was maintained at pH 12 using 5 M NaOH. For the 60 °C condition, the solution was stirred continuously at 60 °C for 2 h on a magnetic stirrer hotplate until a white precipitate formed. For the room temperature (28 °C) synthesis, the solution was treated similarly without heating. The precipitate was centrifuged at 15,000 rpm for 5 min and washed twice with distilled water. The precipitate was further dried at room temperature (28 °C) overnight [2].

### 2.4. Characterization ZnO NPs

The functional groups of the ZnO NPs were characterized by Fourier transform infrared (FTIR) spectroscopy in the frequency range from 400 to 4000 cm^−1^ using a Thermo Nicolet Nexus FTIR spectrophotometer (Smart Orbit, Woodland, CA, USA). The FTIR spectra of the samples were also recorded on a 1752X spectrophotometer (Perkin Elmer, Waltham, MA, USA) using the KBr disc method.

X-ray diffraction (XRD) patterns were obtained with a PXRD-6000 diffractometer (Shimadzu, Japan) with a dwell time of 0.5°/min. Data were recorded in the 2*θ* range from 4° to 90° (diffraction angle 2*θ*) using Cu-Kα radiation (λ = 1.5418 Å) generated at 30 kV and 30 mA. For XRD sample preparation, 10 g of sample was ground to a fine powder and deposited into a hollow space in the middle of a flat sample-holder plate. To achieve a random distribution of lattice orientations of the sample, the upper surface of the sample was well-flattened and the XRD pattern was recorded. The same procedure was repeated for all the samples in the study.

The atomic percentage or ratio of O to Zn (O:Zn) and the surface morphology of the samples were determined at the Institute of Bioscience and Institute of Advanced Technology, Universiti Putra Malaysia via field-emission scanning electron microscopy (FESEM) and transmission electron microscopy (TEM). The sample was placed on a stub and examined at 5 kV using a JSM-7600F (JEOL, Tokyo, Japan) field emission scanning electron microscope. The TEM micrographs were obtained using a H-7100 (Hitachi, Tokyo, Japan) or a Tecnai G2 (FEI, Hillsboro, OR, USA) by placing a droplet of sonicated dispersion of the sample directly onto a carbon-coated copper grid (mesh size 300) that was subsequently dried at 37 °C for 24 h.

### 2.5. Preparation of ZnO–Starch Nanocomposite Films

The preparation of ZnO–starch packaging films via the casting method was based on the method of Alebooyeh et al. [72] with some modifications. The ZnO NP powder at different concentrations (1%, 3%, and 5% w/w dry basis) was dispersed in 50 mL of distilled water and stirred using magnetic stirrer hotplate at room temperature (28 °C) for 60 min. The dispersing solution was sonicated using an ultrasonicator (QSonica, Newton, CT, USA) operating at 500 W and 20 kHz, for 30 min with a sequence of 1 min of sonication and 10 s of rest, at an amplitude of 50% to ensure good dispersion.

The starch solution was prepared by a casting method whereby 3 g of cassava starch was dispersed in 100 mL of distilled water solution to attain a 3% w/w suspension. Glycerol at a concentration of 40% w/w of the dry starch was added as a plasticizer [73]. The solution was heated with continuous stirring using a magnetic stirrer until gelatinized completely at 75 °C. The starch solution was then cooled to 40 °C before being mixed with the ZnO NP suspension (refer to Figure S1).

The ZnO–starch nanocomposite film of solution was prepared by mixing 50 mL of the ZnO NP suspension with 100 mL of gelatinized starch solution and stirring the resultant mixture for 30 min using magnetic stirrer hotplate. The solution was then sonicated for 5 min to produce a homogenous solution. Then, 25 mL of the solution were poured into an acrylic Petri dish (diameter: 14 cm) and left in an air-conditioned room (20 °C) for 48 h on a flat table. A neat starch film without ZnO NPs was also prepared as the control film. After drying, the film was peeled and conditioned in a desiccator containing saturated MgNO_3_ solution (RH: 51%, temperature: 30 °C) in a sealed plastic bag [74].

### 2.6. Antibacterial Activity Assay

Antibacterial activity of the ZnO–starch films was investigated on Gram-negative bacteria *Salmonella enterica* serovar Choleraesuis and Gram-positive bacteria *Bacillus subtilis* UPMC1175. The antibacterial activity was investigated using the disc diffusion method and the bacterial growth was monitored by measurement of optical density (OD) of solution compared with a 0.5 McFarland standard solution and estimation of colony forming units (CFUs) on solid growth. The bacterial isolates were cultured on nutrient broth (Merck, Darmstadt, Germany) at 37 °C overnight. Following incubation, a standard inoculum of each bacterial isolate was prepared in sterile normal saline to a concentration of 1.5 × 10^8^ CFU/mL and compared with a 0.5 McFarland standard solution. A sterile swab was dipped into the suspension and then inoculated on Muller–Hinton agar (Merck, Darmstadt, Germany) plate to provide uniform coverage of bacteria on the surface of the plate. The ZnO composite films with 6 mm diameter containing 1%, 3%, and 5% ZnO NPs and a negative control film (without ZnO NPs) were placed onto the plates. Streptomycin antibiotic (100 mg/mL) was pipetted onto the film disc, which acted as a positive control (labeled as “+ve” for the studies. The plates were incubated at 37 °C for 24 h; after incubation, the diameter of the inhibition zone of each plate was examined.

## 3. Results and Discussion

### 3.1. Functional Analysis

**Figure 1:**
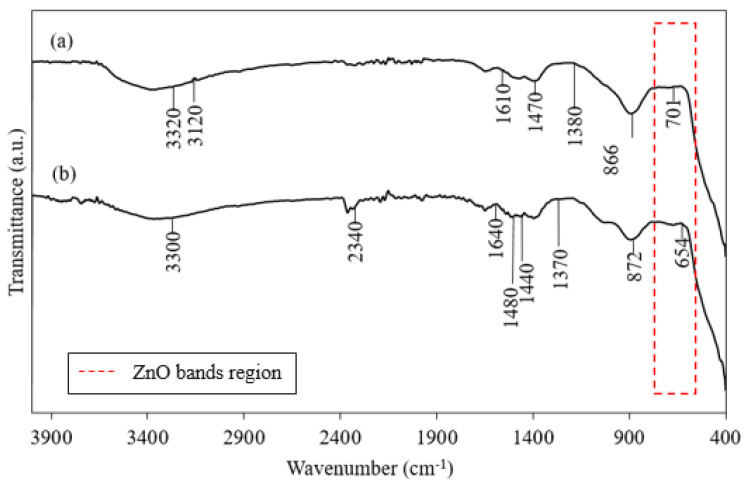
FTIR spectrum of synthesized ZnO NPs prepared (**a**) with heating (60 °C) and (**b**) without heating (28 °C).

The FTIR spectra of the ZnO NPs synthesized with heating at 60 °C and without heating (28 °C) are shown in Figure 1. The spectrum of the heated ZnO NPs (Figure 1a) showed absorption bands at 3320, 3120, 1610, 1470, 1380, 866, and 701 cm^−1^. The FTIR spectrum of the green-synthesized ZnO NPs obtained without heating (Figure 1b) showed bands at 3300, 2340, 1640, 1480, 1440, 1370, 872, and 654 cm^−1^.

Table 1 shows the assignments of the different peaks in the spectra of ZnO synthesized under different conditions. The FTIR spectra show a broad band at 3320 to 3120 cm^−1^ because of O–H functional groups on the ZnO NPs produced under both conditions. The aldehyde C–H stretching band at 2340 cm^−1^ was observed only in the spectrum of the ZnO NPs prepared under the non-heated condition. The bands at 1640 to 1610 cm^−1^ were attributed to the C=O stretching vibration of carboxylic acid groups. The absorption bands at 1480 to 1470 cm^−1^ corresponded to the NH stretching vibration of amine groups. The peak in the range 1440–1380 cm^−1^ corresponds to a CH_3_ bending vibration. The peaks in the region below 1000 cm^−1^, which is known as the fingerprint region, characterize the intrinsic adsorption bands of metal oxides [75]. The ZnO absorption band appeared in the range from 800 to 400 cm^−1^ [76], which is marked with a red dashed line box.

### 3.2. XRD Analysis

**Figure 2:**
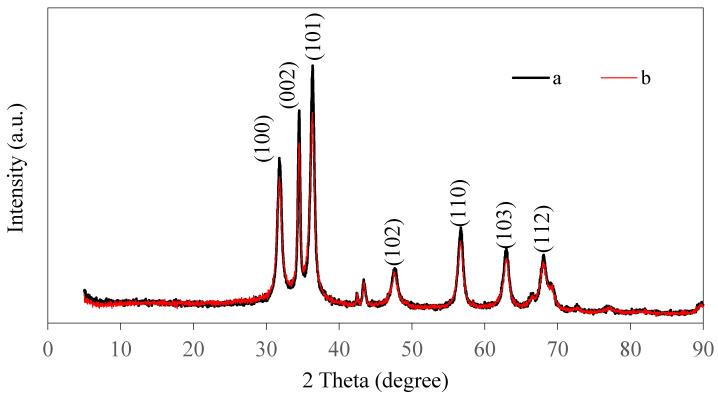
XRD pattern of ZnO NPs synthesized (**a**) with heating (60 °C) and (**b**) without heating (28 °C).

The ZnO NPs prepared from pineapple peel were characterized using Cu-Kα XRD to confirm the formation of ZnO NPs and analyze their structure (Figure 2). The peaks in the pattern of the ZnO NPs synthesized with heating were observed at 31.87°, 34.47°, 36.34°, 47.73°, 56.66°, 62.96°, and 68.28°, which correspond to the (100), (002), (101), (102), (110), (103), and (112) planes of ZnO, respectively. These peaks were similar to those reported by Kumar et al. [78] and also by JCPDS file No. 36-1451. Similarly, the diffraction peaks in the pattern of the ZnO NPs prepared without heating were observed at 31.80°, 34.32°, 36.29°, 47.53°, 56.64°, 62.33°, and 67.99°. These diffraction peaks were also similar to those detailed by Talam et al. [79], who reported diffraction peaks at 31.84°, 34.52°, 36.33°, 47.63°, 56.71°, 62.96°, and 68.13°. The XRD patterns show that the ZnO NPs synthesized with and without heating crystallize in the hexagonal wurtzite structure. The sharp and intense peaks indicate that the structures of the synthesized ZnO NPs were highly crystalline. Similar results have been reported by Suresh et al. [3] and Ramesh et al. [76] for ZnO NPs.

### 3.3. EDX Analysis

**Figure 3:**
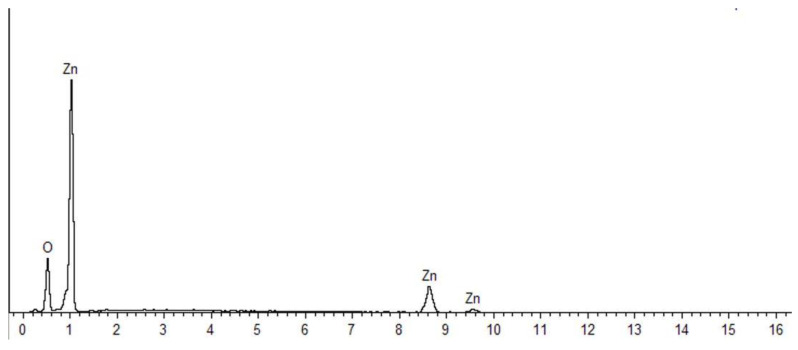
Energy-dispersive X-ray spectrum of ZnO NPs synthesized with heating (60 °C).

**Figure 4:**
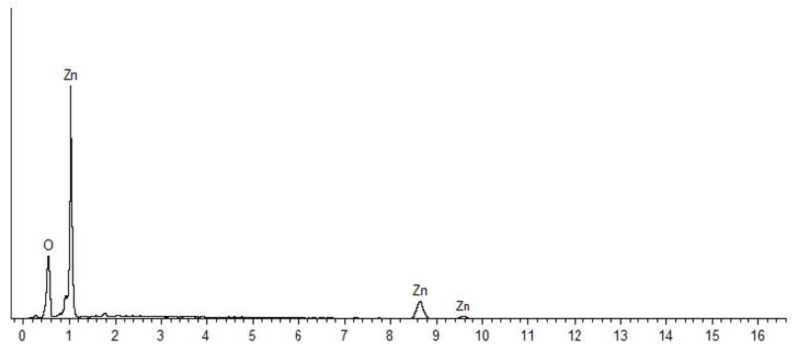
Energy-dispersive X-ray spectrum of ZnO NPs synthesized without heating (28 °C).

Figure 3 and Figure 4 shows the EDX spectra of the synthesized ZnO NPs, with strong signals for Zn and O atoms, confirming the purity of the samples. The atomic and weight percentages of Zn and O were 46.34% (77.92%) and 53.66% (22.08%) for NPs synthesized at 60 °C (Figure 3), respectively. By contrast, the NPs prepared without heating exhibited atomic and weight percentages of Zn and O of 42.54% (75.15%) and 57.46% (24.85%) (Figure 4), respectively.

### 3.4. FESEM and TEM Analyses

**Figure 5:**
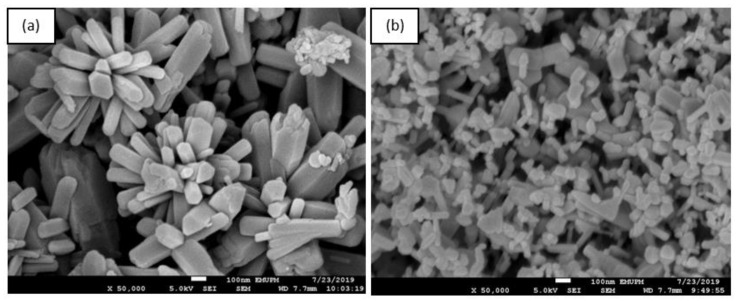
FESEM images of synthesized ZnO NPs observed at 50,000×: (**a**) heated (60 °C) and (**b**) non-heated (28 °C).

The FESEM analysis was conducted to determine the structure of the obtained reaction products. The images show individual particles as well as several aggregates. Figure 5a,b shows the structure of ZnO NPs synthesized at different temperatures (28 °C and 60 °C). The ZnO NPs synthesized at 60 °C (Figure 5a) exhibit flower-rod shapes as compared with the ZnO NPs prepared at 28 °C, which consist of a mixture of spherical and rod-shaped particles (Figure 5b). The ZnO NPs prepared at 28 °C were clearly a better separated and less agglomerated mixture of spherical- and rod-shaped particles compared with the NPs prepared at 60 °C, which agglomerated to form flower-rod-shaped structures. The flower-shaped particles tend to stick to one another, thus forming large agglomerates.

**Figure 6:**
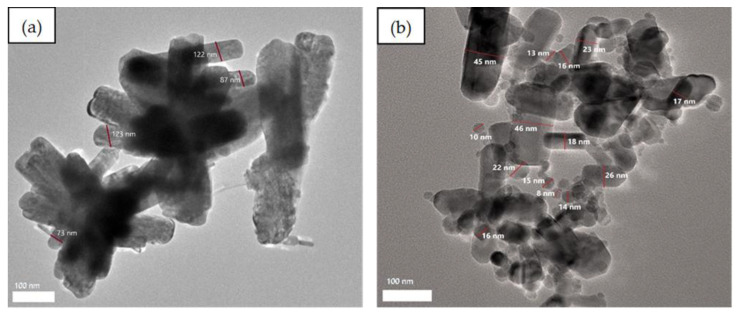
TEM images of synthesized ZnO NPs: (**a**) heated (60 °C) and (**b**) non-heated (28 °C).

According to Kumar et al. [80], the growth of the ZnO NPs’ flower-rod-shaped structure is due to the addition of sodium hydroxide (NaOH) to zinc hexahydrate, which leads to the formation of zincate ions. When the solution containing zincate ions is heated, it slowly converts into hydroxyl ions and ZnO. This phenomenon occurs because the ZnO crystal structure is constructed gradually by OH^−^ ions and acts as a polar crystal. When the particles become saturated, the ZnO nucleus grows, resulting in ZnO rods. Over time, freshly formed nanorods deposited onto the surface of previously formed crystallites lead to the formation of leaves as the first structure; these leaves eventually coalesce into various flower-shaped ZnO NPs. This process was promoted by the heat treatment, which provided sufficient heat energy for atom nucleation, which led to enhanced surface mobility and a more active surface. The TEM micrographs show that smaller ZnO NPs were obtained at room temperature, where the diameter was in the range 8–46 nm (Figure 6b), than at 60 °C, where the diameter was in the range 73–123 nm (Figure 6a). According to Shaziman et al. [81], ZnO NPs grew less densely packed with increasing temperature. An increase in reaction temperature can enhance the aggregation of ZnO NPs as well as increase the frequency of collisions among nucleating atoms during synthesis. These collisions cause nucleated atoms to disperse, which quickly increases surface mobility and makes them more active, reducing the formation of tight aggregates. The synthesis of nanostructured zinc oxide particles using chemical and biological method, had produced highly stable and spherical zinc oxide [46].

### 3.5. Antibacterial Activity

Antibacterial activity of the ZnO NPs incorporated into starch films was investigated using food-borne pathogens: Gram-negative *Salmonella enterica* serotype Choleraesuis and Gram-positive *Bacillus subtilis*. The presence of an inhibition zone surrounding the composite film indicates that ZnO NPs are anti-bacterially active, where the mechanism of antibacterial activity of ZnO NPs involves disrupting the membrane structure of the bacteria [39].

**Figure 7:**
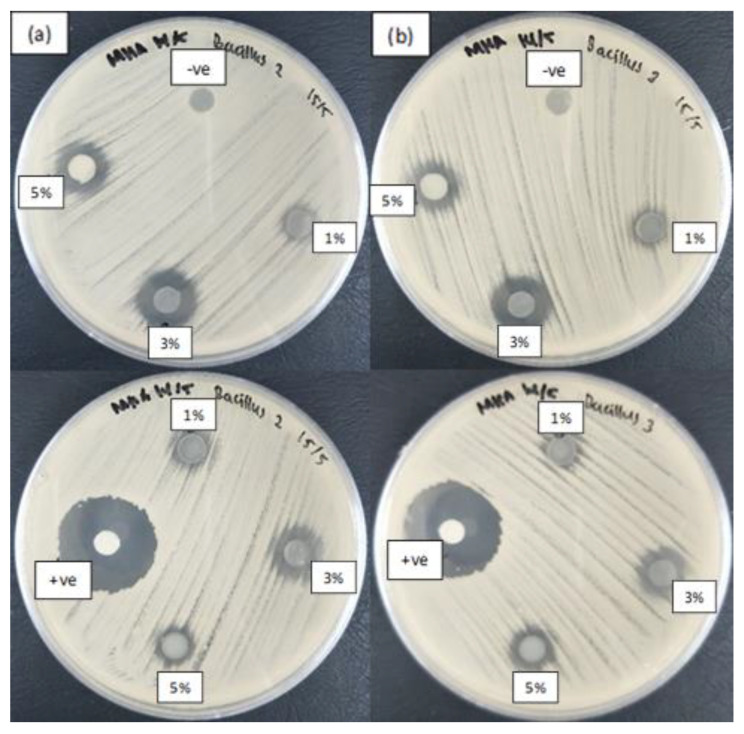
Zone of inhibition of starch films incorporated with different concentrations of synthesized ZnO NPs synthesized at column (**a**) with heating (60 °C) and column (**b**) without heating (28 °C) against Gram-positive *Bacillus subtilis UPMC 1175*. Film without ZnO NPs acted as negative control (“-ve”), while streptomycin antibiotic as positive control (“+ve”).

**Figure 8:**
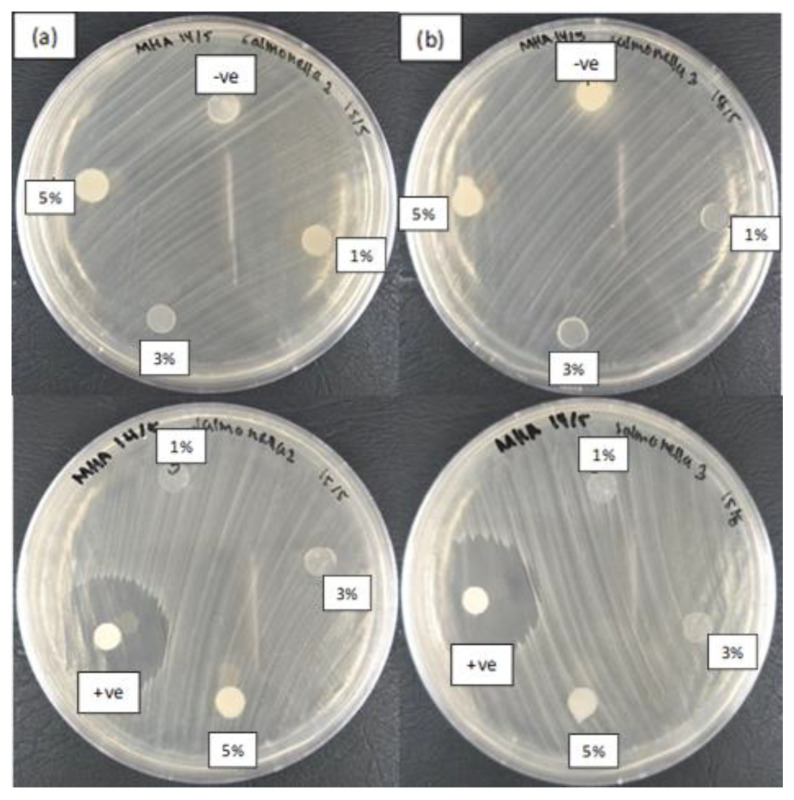
Zone of inhibition of starch films incorporated with different concentrations of synthesized ZnO NPs synthesized at column (**a**) with heating (60 °C) and column (**b**) without heating (28 °C) against Gram-negative *Salmonella enterica* serotype Choleraesuis. Film without ZnO NPs acted as negative control (“-ve”), while streptomycin antibiotic as positive control (“+ve”).

According to the results in Figure 7, both the ZnO NPs synthesized with heating and those synthesized without heating demonstrated antibacterial activity toward Gram-positive bacteria *B. subtilis* but not toward on Gram-negative *Salmonella enterica* serotype Choleraesuis (Figure 8). This finding indicates that Gram-positive bacteria are more susceptible to the synthesized ZnO NPs than Gram-negative bacteria. These results are consistent with those of Hajipour et al. [82], who reported a similar trend Gram-positive bacteria are more sensitive to ZnO NPs than Gram-negative bacteria because the cell walls in Gram-positive bacteria render them more susceptible to interaction with ZnO NPs [83]. This effect is evident through observation of the different wall structures of these two types of bacteria. Gram-positive bacteria have a simpler structure than Gram-negative bacteria, which are known for their complex cell wall structure; Gram-negative bacteria thus provide greater resistance toward antibacterial agents. Gram-negative bacteria are surrounded by additional outer membranes composed of lipopolysaccharides, which provide an extra layer of protection and resistance toward antibacterial agents because of the presence of negative charges. These structures increase the thickness of the cell wall and prevent the ZnO NPs from penetrating into the cells. Thus, the cell walls provide a barrier to ZnO NPs, preventing them from interacting with the internal components of the cells.

Figure 7a also shows a smaller inhibition zone for the ZnO NPs synthesized at 60 °C than for those synthesized at 28 °C. This difference might be due to the agglomeration of ZnO NPs, as supported by the particle size distributions in Figure 5 and Figure 6. The images show that ZnO NPs synthesized at 60 °C exhibited high agglomeration, resulting in a larger particle size, which made them unable to penetrate the cell membrane of bacteria and caused a reduction in antibacterial activity.

In addition to the types of bacteria, the concentration of ZnO NPs incorporated into the film also played a critical role in determining its antibacterial activity. Inhibition diameters of 9.67 and 15 mm were observed on non-heated ZnO NP–starch films incorporated with 1% and 3% NPs, respectively, whereas the inhibition diameters were 9 and 13 mm on the heated ZnO NP–starch films (Table 2). These results show that, for ZnO NPs prepared at both temperatures, the zone of inhibition for *B. subtilis* tended to increase with the increasing concentration.

Although an increase in the concentration of ZnO NPs was expected to increase the diameter of the zone of inhibition, the results in this study were not strictly consistent with the expected results. The susceptibility of the bacterial strain toward ZnO NPs was limited to a specific concentration because a further increase in concentration to 5% reduced the antibacterial activity of ZnO–starch film (Table 2). An increase in the zone of inhibition was observed when the concentration was increased from 1% to 3%, but a decrease was observed when the concentration of ZnO NPs was increased to 5%. This result is attributable to the size of the NPs. Particle size strongly influences antibacterial activity. Tayel et al. [61], who conducted studies on the antibacterial activity of ZnO against several bacterial strains, found that nanosized ZnO particles exhibit greater antibacterial activity than micron-sized ZnO particles. Thus, ZnO NP concentrations of 1% and 3% are considered sufficient concentrations for incorporation into starch films to inhibit bacterial growth because a higher percentage may induce agglomeration of the NPs, thereby reducing their antibacterial activity. The antibacterial effect of the metal oxide nanoparticles incorporated in composite films might be released in the form of metal ions into a physiological solution and direct contact of bacteria with the metal oxide nanocomposite film [84,85].

## 4. Conclusions

The ZnO NPs were successfully synthesized from pineapple peel extract which acted as a natural reducing agent in the bioreduction process. The FTIR, EDX, XRD, FESEM, and TEM showed differences in particle structure, size, morphology, and chemical constituent of ZnO NPs synthesized at 60 °C and 28 °C. The synthesis of ZnO NPs at 60 °C resulted in a mixture of spherical- and rod-shaped structures, whereas the synthesis at 28 °C resulted in spherical flower-shaped structures. The synthesis temperature of 60 °C was found to produce the smallest ZnO NPs. The green synthesis method adopted in this work is simple and environmentally friendly because it generates little residue and contaminants while still leading to ZnO NPs. Heat treatment of ZnO NP–starch films at 1%, 3%, and 5% ZnO NPs exhibited good antibacterial activity against a Gram-positive bacteria, *Bacillus subtilis*. This study conclusively reports an eco-friendly approach for the synthesis of ZnO NPs, whereby the ZnO NPs could be incorporated into a film for use in food packaging to inhibit the growth of bacteria.

## Figures and Tables

**Table 1:** Assignment of different peaks in the FTIR spectra of ZnO NPs prepared under heating (60 °C) and non-heating (28 °C) conditions.

Wavenumbers	Heating (60 °C)	Non-Heating (28 °C)		Peak Assignment	Reference
3330–3120	+		+	O–H stretching band	[77]
2340			+	C–H stretching	[77]
1640–1610	+		+	C=O functional group	[2]
1480–1470	+		+	Amine NH vibration stretch	[2]
1440–1380	+		+	CH_3_ bending	[68]
872–866	+		+	Absorption band of ZnO	[76]
701–654	+		+		

Note: Symbol “+” indicates the presence of the assigned peak.

**Table 2:** Antibacterial activity of synthesized ZnO NPs against Gram-positive and Gram-negative bacteria, measured as the diameter of the inhibition zone.

Concentration of ZnO NPs in Starch Films		Zone of Inhibition (mm)							
		*Bacillus subtilis UPMC 1175*				*Salmonella enterica* serotype Choleraesuis			
		i	ii	iii	Mean	i	ii	iii	Mean
Nonheated (28 °C)	0%	Inhibition Zone Diameter				No Inhibition Zone			
	1%	11	9	9	9.67				
	3%	15	15	15	15.00				
	5%	12	13	12	12.33				
Heated (60 °C)	1%	9	9	8	8.67				
	3%	13	13	14	13.33				
	5%	10	11	12	11.00				

The inhibition zone (mm) includes the film sample assay disc diameter (6 mm). The diameter of the inhibition zone is the mean of triplicate measurements.

## Supplementary Materials

The following are available online at https://www.mdpi.com/2079-4991/10/6/1061/s1, Figure S1: Photo image of starch film (0 wt.% ZnO which acts as a control film).
