# Supplementary material for: Effect of Synthesis Temperature on the Size of ZnO Nanoparticles Derived from Pineapple Peel Extract and Antibacterial Activity of ZnO–Starch Nanocomposite Films
Source: Nanomaterials (Basel). 2020 May 30;10(6):1061. doi: 10.3390/nano10061061 (PMC7352361; doi:10.3390/nano10061061)
Supplement: Supplementary file 1 [file nanomaterials-10-01061-s001.pdf]

# Supplementary Materials:

# Effect of Synthesis Temperature on the Size of ZnO Nanoparticles Derived from Pineapple Peel Extract and Antibacterial Activity of ZnO–Starch Nanocomposite Films

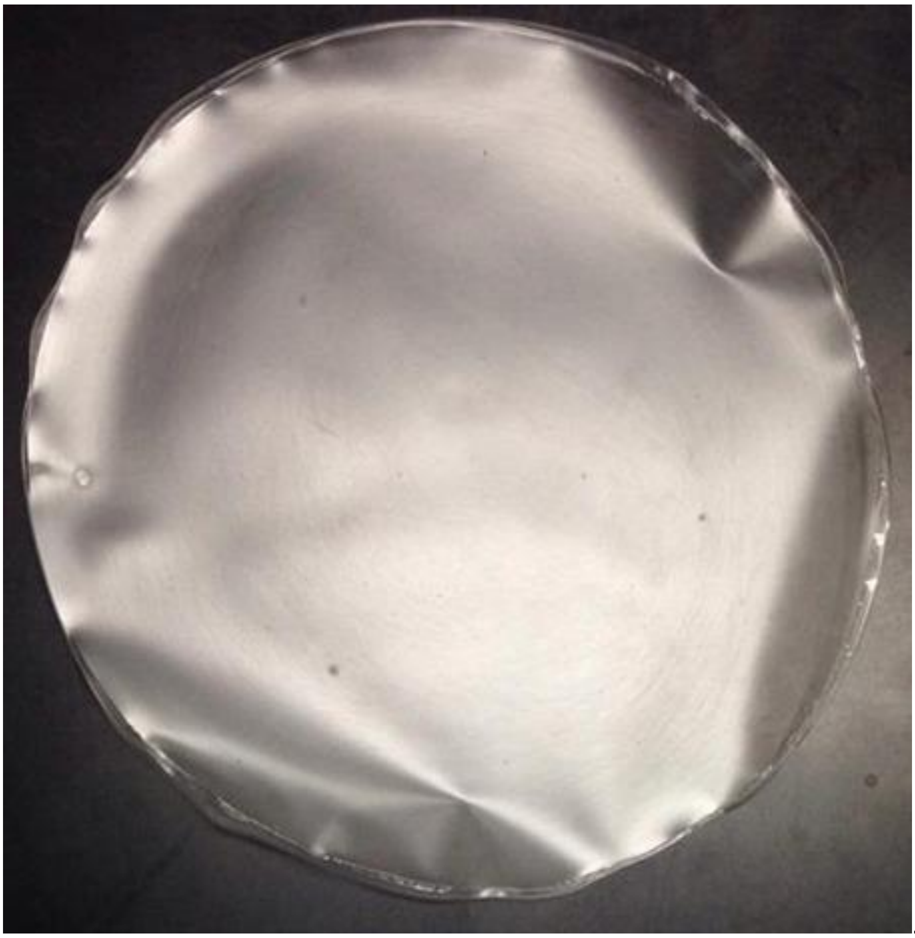

**Figure S1.** Photo image of starch film (0 wt.% ZnO which acts as a control film).
